# The role of MAPK11/12/13/14 (p38 MAPK) protein in dopamine agonist-resistant prolactinomas

**DOI:** 10.1186/s12902-021-00900-9

**Published:** 2021-11-23

**Authors:** Shuman Wang, Aihua Wang, Yu Zhang, Kejing Zhu, Xiong Wang, Yonggang Chen, Jinhu Wu

**Affiliations:** 1 Central lab, Tongren Hospital Affiliated to Wuhan University, The Third Hospital of Wuhan, 241 Pengliuyang Road, Wuchang District, Wuhan, 430060 Hubei China; 2 Department of Pharmacy, Tongren Hospital Affiliated to Wuhan University, The Third Hospital of Wuhan, Wuhan, 430060 Hubei China; 3 College of Pharmacy, Hubei University of Chinese Medicine, Wuhan, 430065 Hubei China; 4 Health Examination Center, Tongren Hospital Affiliated to Wuhan University, The Third Hospital of Wuhan, 241 Pengliuyang Road, Wuchang District, Wuhan, 430060 Hubei China

**Keywords:** Prolactinomas, Dopamine agonist, Bromocriptine, Drug resistance, MAPK11/12/13/14

## Abstract

**Background:**

Prolactinoma is a functional pituitary adenoma that secretes excessive prolactin. Dopamine agonists (DAs) such as bromocriptine (BRC) are the first-line treatment for prolactinomas, but the resistance rate is increasing year by year, creating a clinical challenge. Therefore, it is urgent to explore the molecular mechanism of bromocriptine resistance in prolactinomas. Activation of the P38 MAPK pathway affects multidrug resistance in tumours. Our previous studies have demonstrated that inhibiting MAPK14 can suppress the occurrence of prolactinoma, but the role of MAPK11/12/13/14 (p38 MAPK) signalling in dopamine agonist-resistant prolactinomas is still unclear.

**Methods:**

A prolactinoma rat model was established to determine the effect of bromocriptine on MAPK11/12/13/14 signalling. DA-resistant GH3 cells and DA-sensitive MMQ cells were used, and the role of MAPK11/12/13/14 in bromocriptine-resistant prolactinomas was preliminarily verified by western blot, RT-qPCR, ELISA, flow cytometry and CCK-8 experiments. The effects of MAPK11 or MAPK14 on bromocriptine-resistant prolactinomas were further verified by siRNA transfection experiments.

**Results:**

Bromocriptine was used to treat rat prolactinoma by upregulating DRD2 expression and downregulating the expression level of MAPK11/12/13/14 in vivo experiments. The in vitro experiments showed that GH3 cells are resistant to bromocriptine and that MMQ cells are sensitive to bromocriptine. Bromocriptine could significantly reduce the expression of MAPK12 and MAPK13 in GH3 cells and MMQ cells. Bromocriptine could significantly reduce the expression of MAPK11, MAPK14, NF-κB p65 and Bcl2 in MMQ but had no effect on MAPK11, MAPK14, NF-κB p65 and Bcl2 in GH3 cells. In addition, knockdown of MAPK11 and MAPK14 in GH3 cells by siRNA transfection reversed the resistance of GH3 cells to bromocriptine, and haloperidol (HAL) blocked the inhibitory effect of bromocriptine on MAPK14, MAPK11, and PRL in MMQ cells. Our findings show that MAPK11 and MAPK14 proteins are involved in bromocriptine resistance in prolactinomas.

**Conclusion:**

Bromocriptine reduces the expression of MAPK11/12/13/14 in prolactinomas, and MAPK11 and MAPK14 are involved in bromocriptine resistance in prolactinomas by regulating apoptosis. Reducing the expression of MAPK11 or MAPK14 can reverse bromocriptine resistance in prolactinomas.

## Background

Prolactinomas are among the most common intracranial tumours. Prolactin secretion can lead to menstrual and lactation disorders in women and a decline in sexual function in men, and the incidence in women is significantly higher than that in men. Prolactin microadenomas with clinical symptoms generally do not develop into macroadenomas, and some prolactinomas are aggressive, with enlarged tumours and elevated blood prolactin levels [1]. Dopamine agonists (DAs), such as bromocriptine, are first-line treatment drugs. Most prolactinoma patients show a good response to bromocriptine treatment, but 20% of patients display serious drug resistance. The patients took bromocriptine for 3 months, but their serum prolactin did not return to normal levels, and the tumour volume was reduced by less than 50% [2–4]. DA resistance in prolactinoma patients is becoming increasingly serious and severely affects human reproductive health. Therefore, it is a clinical problem that urgently needs to be resolved.

Prolactinoma cells express DA receptors, and DA(s) can effectively inhibit prolactin secretion and shrink tumours by binding to DA receptors on the surface of most patients’ cells. As a result, a decrease in D2 receptor expression levels will cause the failure of DAs to inhibit prolactin and further lead to DA resistance in prolactinoma [5]. Studies have shown that the reduction of dopamine D2 receptor expression is considered to be the main cause of DA resistance in prolactinoma [6–8], but DRD2-mediated resistance cannot explain all the issues. Therefore, it is urgent to explore the mechanism of drug resistance in prolactinoma.

Mitogen-activated protein kinases (MAPKs) include the MAPK/ERK family or classical pathway and the Big MAP kinase-1 (BMK-1), c-Jun N-terminal kinase (JNK), and p38 signalling families. The activation cascade occurs in the following order: MAPKKK (mitogen-activated protein kinase kinase kinases, represented by RAF and its variants), followed by MAPK kinase (MAPKK: MEK1/2/3/4/5/6/7), and finally MAPK. The mitogen-activated protein kinase (MAPK) cascade is a critical pathway for human cancer cell survival, dissemination, and resistance to drug therapy [9, 10]. p38 MAPK includes isoforms p38α, p38β, p38γ, and p38δ. Mammalian p38 kinases share more than 60% amino acid sequence identity, with p38α being 75% identical to p38β and p38γ being 75% identical to p38δ. Moreover, p38α and p38β are ubiquitously expressed, p38α usually at higher levels than p38β except in some brain regions, whereas p38γ and p38δ expression tends to be more tissue-specific, p38γ (MAPK12/ERK6) in muscle, and p38δ (MAPK13/SAPK4) in lung and kidney [11].p38 demonstrates distinct and even opposing effects in different cancers, as it was shown to serve either as a tumor suppressor or tumor promoter. It was also shown that in some cases, it can perform both activities in different stages of cancer development. Although all p38 isoforms have been implicated in the processes listed above, they can be divided into two somewhat distinct subgroups: p38α and p38β (p38α/β) versus p38γ and p38δ. p38β is very similar in amino acid sequence to p38α. They have similar substrate specificities and are sensitive to the same chemical inhibitors, suggesting that p38β and p38α may have overlapping functions [12–14]. Indeed, p38α/β were implicated in the induction and maintenance of several pathologies such as inflammation, cancer, and autoimmune diseases, but also hypertrophy, hypoxic nephropathy, and diabetes. In many cases, the role of p38α/β is not direct, but it is mediated by p38α/β-regulated inflammation, which in turn contributes to the development of the diseases [15]. It is now clear that p38γ and p38δ play crucial roles in inflammation, the development of insulin resistance, neurotoxicity, cell growth, and the progression of tumour formation. Abnormal activity and dysregulation of the p38α/β cascade are associated with a variety of diseases [16].. Activation of the p38 MAPK signalling pathway promotes the occurrence and progression of various tumours, which is responsible for the signal transduction of many chemotherapy drugs and is a necessity for multidrug resistance induced by anticancer drugs in tumour cells. Inhibition of p38 can increase the sensitivity of cisplatin-induced apoptosis in human lung cancer cells and colon cancer cells. Compared with cisplatin alone, the combined administration of cisplatin and p38 inhibitors can significantly inhibit tumour cell proliferation and induce apoptosis [17, 18]. p38 MAPK is involved in cell apoptosis, and the transcription factor NF-κB prevents cell apoptosis by inducing the expression of several antiapoptotic proteins. Changes in genes and proteins that control cell apoptosis are one of the mechanisms underlying multidrug resistance in tumours [19–23]. The p38 MAPK signalling pathway plays an important role in the occurrence and development of the pituitary, and there are multiple potential targets in its treatment [24]. Our previous study found that MAPK14 is highly expressed in prolactinoma and can be suppressed by the inhibition of MAPK14 [25]. The mechanism of MAPK11/12/13/14 in bromocriptine resistance in prolactinoma has not been investigated. Therefore, we studied the effects of bromocriptine on MAPK11, MAPK12, MAPK13, and MAPK14 in rat prolactinoma, compared the difference in the effect of bromocriptine on MAPK11, MAPK12, MAPK13, MAPK14, NF-κB, p65, Bcl2, and Bax in DA-resistant GH3 cells and DA-sensitive MMQ cells, and further evaluated the therapeutic effects of bromocriptine after the reduction of p38 MAPK in GH3 cells. These findings clarify the exact mechanism of the four p38 MAPK subunits in bromocriptine-resistant prolactinomas.

## Materials and methods

### Reagents and antibodies

Bromocriptine mesylate (BRC) (cat. no. HY-12705A) and Haloperidol (cat. no. HY-14538) were purchased from MedChemExpress. Bromocriptine Mesilate Tablets (BRC)(lot. no. T03029A) was purchased from Gedon Richter Plc. Oestradiol benzoate injection was purchased from Sichuan Jinke Pharmaceutical Co., Ltd. Anti-dopamine D2 receptor (D2R; cat. no. 55084–1-AP), MAPK11 (cat. no. 17376–1-AP), MAPK12 (cat. no. 20184–1-AP), MAPK13 (cat. no. 10217–1-AP), MAPK14 (cat. no. 14064–1-AP), Bcl2 (cat. no. 26593–1-AP) and Bax (cat. no. 50599–2-lg) primary antibodies were purchased from Proteintech. β-actin (cat. no. T0022), PRL (cat. no. DF6506) and NF-κB p65 (cat. no. BF0382) antibodies were purchased from Affinity. HRP Goat Anti-Mouse lgG (cat. no: AS003) and HRP Goat Anti-Rabbit lgG (cat. no: AS014) secondary antibodies were purchased from ABclonal.

### Cell culture

The MMQ and GH3 rat prolactinoma cell lines were purchased from BeNa Culture Collection. Both MMQ and GH3 cells were derived from rat pituitary tumours. MMQ cells secrete prolactin and express dopamine receptors. GH3 cells can secrete PRL and growth hormone and express oestrogen receptors but lack dopamine receptors or have low dopamine receptor expression. It is an ideal in vitro cell model for studying the formation mechanism of prolactinomas. It is especially suitable for the study of DA-resistant prolactinomas [26–30]. GH3 cells and MMQ cells were cultured in DMEM (Gibco, Thermo Fisher Scientific, Inc.) and DME/F-12 (1:1) medium (HyClone, GE Healthcare Life Sciences) containing 10% foetal bovine serum (FBS, Gibco, Thermo Fisher Scientific, Inc.) and were maintained at 37 °C in a 5% CO2 atmosphere.

### Animal models

At present, the establishment of a prolactinoma animal model mainly includes an oestrogen-induced prolactinoma model and a transgenic animal prolactinoma model. The estrogen-induced prolactinoma model is mainly established by intraperitoneal injection of oestradiol benzoate into rats. With the extension of oestradiol benzoate induction time, the pituitary gland gradually increases, and the serum prolactin level increases. This method not only has high induction efficiency but also has good stability. It is an extremely ideal animal model for studying the pathogenesis and efficacy of prolactinoma [31–34]. Female SD rats (weight 200 ± 20 g) were purchased from Spelford (Beijing) Biotechnology Co., Ltd. First, these SD rats were randomly divided into a control group, model group and BRC group. Rats in the model group and BRC group were intraperitoneally injected with oestradiol benzoate at a dose of 20 mg/kg once every 5 days for 50 days. At this point, the pituitary volume and the serum prolactin content increased, and the prolactinoma rat model was successfully established. Then, in the BRC group, 2 mL of bromocriptine (BRC) solution was intragastrically administered at a dose of 0.9 mg/kg once a day for 30 consecutive days. All animal experiments were approved by the Ethics Committee of Tongren Hospital Affiliated to Wuhan University, The Third Hospital of Wuhan. All rats were raised and housed in SPF-grade cages and were provided with autoclaved food and water.

### Animal sample collection

After bromocriptine administration, all rats were anaesthetized with 1% pentobarbital sodium 40 mg/kg by intraperitoneal injection, and blood was taken from the abdominal aorta, transferred to a clean centrifuge tube, placed at room temperature for 2 h, and centrifuged at 1000×g for 20 min. Then, the upper serum was collected in a clean centrifuge tube and stored at 4 °C. After the rats were sacrificed with 100% carbon dioxide, the pituitary gland was removed and stored at − 80 °C. The sacrificed rats were sent to the hospital experimental animal treatment centre for unified treatment.

### RNA interference

In total, three different sets of small interfering RNA sequences of MAPK14 or MAPK11 were designed and synthesized by Guangzhou RiboBio Co., Ltd. The sequences of the three MAPK14 siRNA sequences used were i) GGTCCCTGGAGGAATTCAA; ii) CCGAAGATGAACTTCGC-AA; and iii) GGACCTCCTTATAGACGAA. The sequences of the three MAPK11 siRNA sequences were as follows: i) GCAATGTAGCAGTGAATGA; ii) CCACATCCATCCATCGAGGATTT; and iii) GCCCTATGATGAAAGTGTT. A total of 2 × 10^5^ GH3 cells/well was seeded into a 6-well plate and transfected with the different MAPK14 siRNA or MAPK11 siRNA separately. The cells were transfected using Lipofectamine®2000 (Invitrogen; Thermo Fisher Scientific, Inc. in Opti-MEM without serum according to the manufacturer’s protocol. After 24 h of transfection, the cells were treated with or without bromocriptine for 48 h, and then the cell protein was extracted.

### Cell viability assay

Cell viability was measured using the Cell Counting Kit-8 (CCK-8) assay (Biosharp, Beijing Labgic Technology Co., Ltd). According to the manufacturer’s instructions, log-phase MMQ and GH3 cells were collected and adjusted to 1 × 10^5^ cells/ml. A 100 μL aliquot of the cell suspension was added to each well in a 96-well plate. After being placed in an incubator for 12 h, the cells were treated with various concentrations of bromocriptine (BRC) dissolved in medium for 48 h. Ten microlitres of the CCK-8 stock solution was added to the media. The optical density values (OD values) were measured at 450 nm by absorbance microplate reader (CMAX PLUS, Molecular Devices). The cell viability and inhibition ratios were calculated by OD values.

### Apoptosis analysis

Apoptosis was detected by flow cytometry with an Annexin V-PE/7-AAD Apoptosis Detection Kit I (cat. no: MA0220, Meilunbio, Thermo Fisher Biochemical Products (Beijing) Co., Ltd.) according to the manual’s instruction. In brief, cells were double-stained with PE Annexin V and 7-AAD for 15 min in the dark at room temperature and then analysed by flow cytometry (CytoFLEX, Beckman).

### Enzyme-linked immunosorbent assay (ELISA)

GH3 and MMQ cells in the logarithmic growth phase were seeded into a 6-well plate at a density of 2 × 10^5^ cells/mL and cultured separately in 10% FBS DMEM and 10% FBS DME/F-12 medium for 12 h. The cells were treated with different concentrations of BRC (12.5 μM, 25 μM, 50 μM, and 100 μM) for 48 h. The supernatant was collected and tested using a Rat PRL (Prolactinoma) ELISA kit (cat. no. E-EL-R0052c, Elabscience, Co., Ltd) from Elabscience. All procedures were conducted following the kit instructions. The detection method of rat serum PRL levels was the same as above.

### Real-time fluorescence quantitative PCR

Total RNA was extracted from tissues or cells using a Total RNA Kit (Omega, cat. no. R6934–1) and reverse-transcribed into cDNAs using the Servicebio RT First Strand cDNA Synthesis Kit (Servicebio, cat. no. G3330–100) according to the manufacturer’s instructions. qPCR was performed using 2xSYBR Green qPCR Master Mix (Low ROx) (Servicebio, cat. no. G3321–05) on an Mx3000P Real-Time PCR system (Agilent Technologies, Inc.) according to the kit instructions. The thermocycling conditions for RT-qPCR were 95 °C for 2 min and 40 cycles of 95 °C for 30 s, 55 °C for 20 s, and 72 °C for 30 s, followed by 95 °C for 1 min and 55 °C for 30 s. The sequences of the rat-primers used were: GAPDH:forward: GACAT-GCCGCCTGGAGAAAC,reverse: AGCCCAGGATGCCCTTTAGT; DRD2: forward: CAGTGAACA-GGCGGAGAATGGATG,reverse: GTGGTGGGATGGATCAGGGAGAG;MAPK11:forward: GCTGA-AGCGGATCATGGAGGTG,reverse: TGGGAGGCAGAGACTGGATGTATG, MAPK12:forward: ATG-CTGGTGTTGGATGCGGAAC,reverse: TCCTCAAGGGTGCGGTCTACG;MAPK13:forward: GGTG-CCGAGTTCGTGCAGAAG,reverse: CAGCATCTTGTCCAGCAGGTCTAC;MAPK14:forward: GCT-GGCTCGGCACACTGATG,reverse: GCCCACGGACCAAATATCCACTG; PRL:forward: GGTTTGG-TCACAACTCCCATCCC,reverse: TGGACAATTTGGCACCTCAGGAAC;Relative gene expression was analysed by the 2^-ΔΔCt^ method and normalized to GAPDH.

### Western blot analysis

Rat pituitary samples and cell samples were lysed in RIPA buffer with protease inhibitors (Roche Diagnostics) for 30 min on ice. Total protein was quantified using a BCA protein concentration determination kit (Wuhan Servicebio Technology Co., Ltd., cat no: G2026-200 T). Equal amounts of protein were separated from 10 to 15% SDS-PAGE gels and transferred onto nitrocellulose membranes. After blocking with 5% milk in TBST for 1 h at room temperature, the membranes were incubated with the aforementioned primary antibodies at 4 °C overnight. The membranes were then washed in TBST buffer and incubated with HRP-conjugated secondary antibodies (1:3000) for 30 min at room temperature. The signals were visualized by a luminescent image analyser (JP-K600, Shanghai Jiapeng Technology Co., Ltd.). Actin was used as a loading control.

### Statistical analysis

GraphPad Prism 8.0 (GraphPad Software, Inc.) version was used for all statistical analysis and graph production.. All values are expressed as the mean ± SEM of at least three independent experiments. Comparisons between difference groups were performed using one-way ANOVA followed by Tukey’s multiple comparisons test. Significant differences were indicated by *p* < 0.05 (^*^p < 0.05, ^**^p < 0.01, ^***^p < 0.001, ^***^p < 0.0001).

## Results

### Bromocriptine treats prolactinoma by upregulating DRD2 levels and downregulating MAPK11/12/13/14 levels in rat prolactinoma

**Fig. 1:**
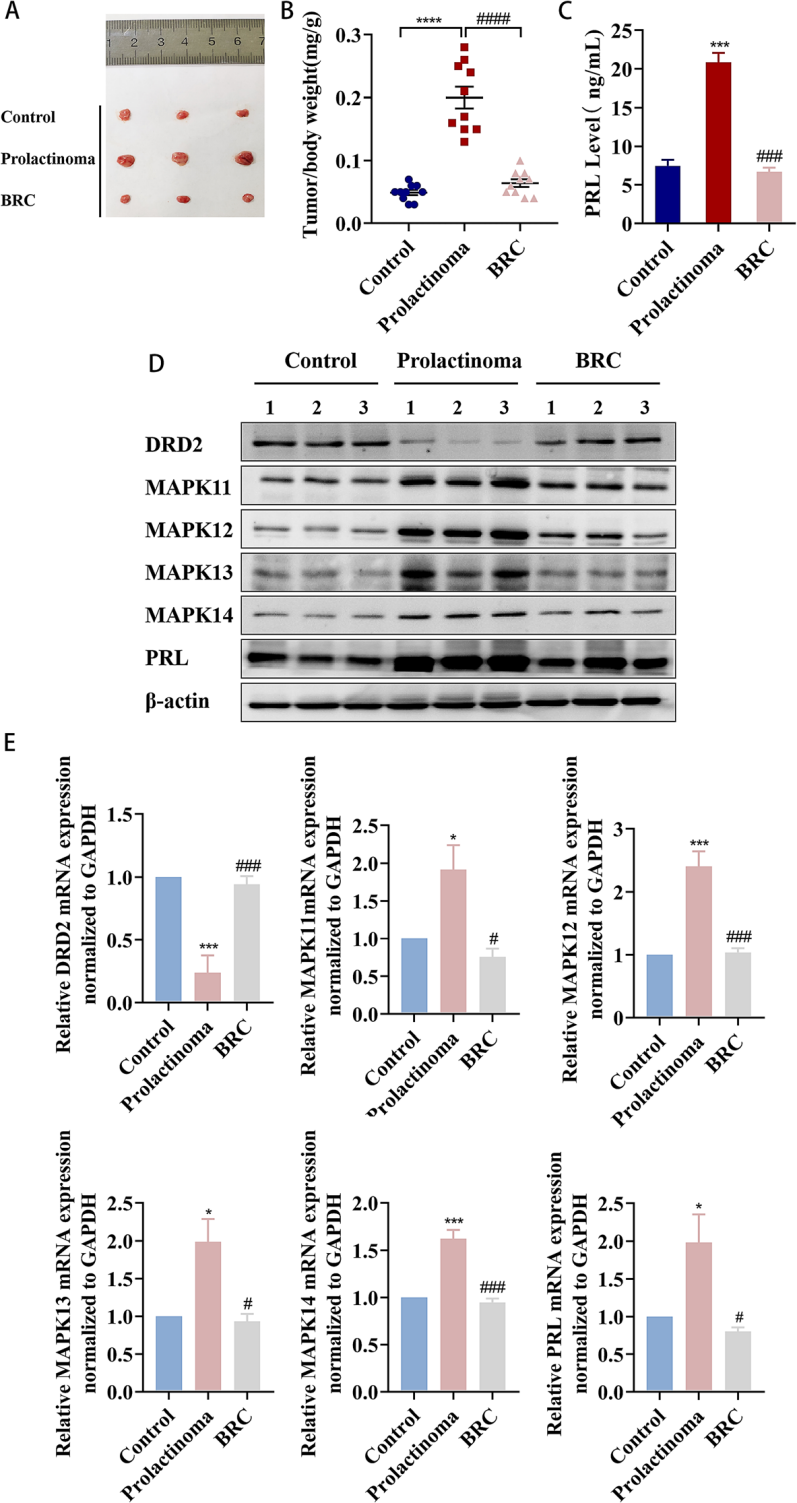
Bromocriptine upregulates DRD2 levels and reduces MAPK11/12/13/14 and PRL levels in rat prolactinomas. **a** Analysis of the appearance of the pituitary in the Control, Prolactinoma, and BRC groups. **b** Ratio of pituitary weight to body weight in rats,^****^P < 0.0001 vs. Control;^####^*P* < 0.0001 vs. Prolactinoma, (*n* = 10). **c** Detection of serum PRL level in rats by ELISA.^***^P < 0.001 vs. Control; ^###^*P* < 0.001 vs. Prolactinoma,(*n* = 6). **d** The protein expression levels of DRD2, MAPK11, MAPK12, MAPK13, MAPK14 and PRL in rat pituitary were detected by western blot. **e** The expression levels of DRD2, MAPK11, MAPK12, MAPK13, MAPK14 and PRL mRNA in rat pituitary were detected by RT-qPCR.^*^p < 0.05 vs. control, ^***^p < 0.001 vs. control, ^#^*p* < 0.05 vs. prolactinoma,^###^*p* < 0.001 vs. Prolactinoma,(*n* = 3)

A prolactinoma rat model was established by oestrogen induction, and the effect of bromocriptine on MAPK11/12/13/14 was studied. We first analysed the appearance of the rat pituitary gland. Compared with the control group, the rat pituitary weight/body ratio in the model group was increased significantly (*P* < 0.0001). Compared with the model group, the rat pituitary-weight/body-weight ratio in the BRC group was decreased significantly (*P* < 0.0001) (Fig. 1a, b). ELISA analysis results showed that bromocriptine (BRC) significantly reduced the level of serum PRL in prolactinoma rats (*P* < 0.001) (Fig. 1c). Western blotting results showed that bromocriptine (BRC) upregulated the expression of DRD2 protein in the pituitary of prolactinoma rats while downregulating the expression of MAPK11/12/13/14 protein (Fig. 1d). In addition, we also used RT-qPCR assays to evaluate the changes in the mRNA levels of DRD2, MAPK11/12/13/14, and PRL in each group, which were consistent with the changes in their protein expression levels. Compared with the control group, the mRNA levels for DRD2 of rat pituitary in the model group were significantly reduced (*P* < 0.001), while the mRNA levels of MAPK11 (*P* < 0.05), MAPK12 (*P* < 0.001), MAPK13 (*P* < 0.05), MAPK14 (*P* < 0.001), and PRL (*P* < 0.05) were significantly increased. Compared with the model group, the mRNA level for DRD2 of rat pituitary in the BRC group was significantly increased (*P* < 0.001), while the mRNA levels of MAPK11 (*P* < 0.05), MAPK12 (*P* < 0.001), MAPK13 (*P* < 0.05), MAPK14 (*P* < 0.001), and PRL (*P* < 0.05) were significantly decreased (Fig. 1e).

### GH3 cells are resistant to bromocriptine and MMQ cells are sensitive to bromocriptine

**Fig. 2:**
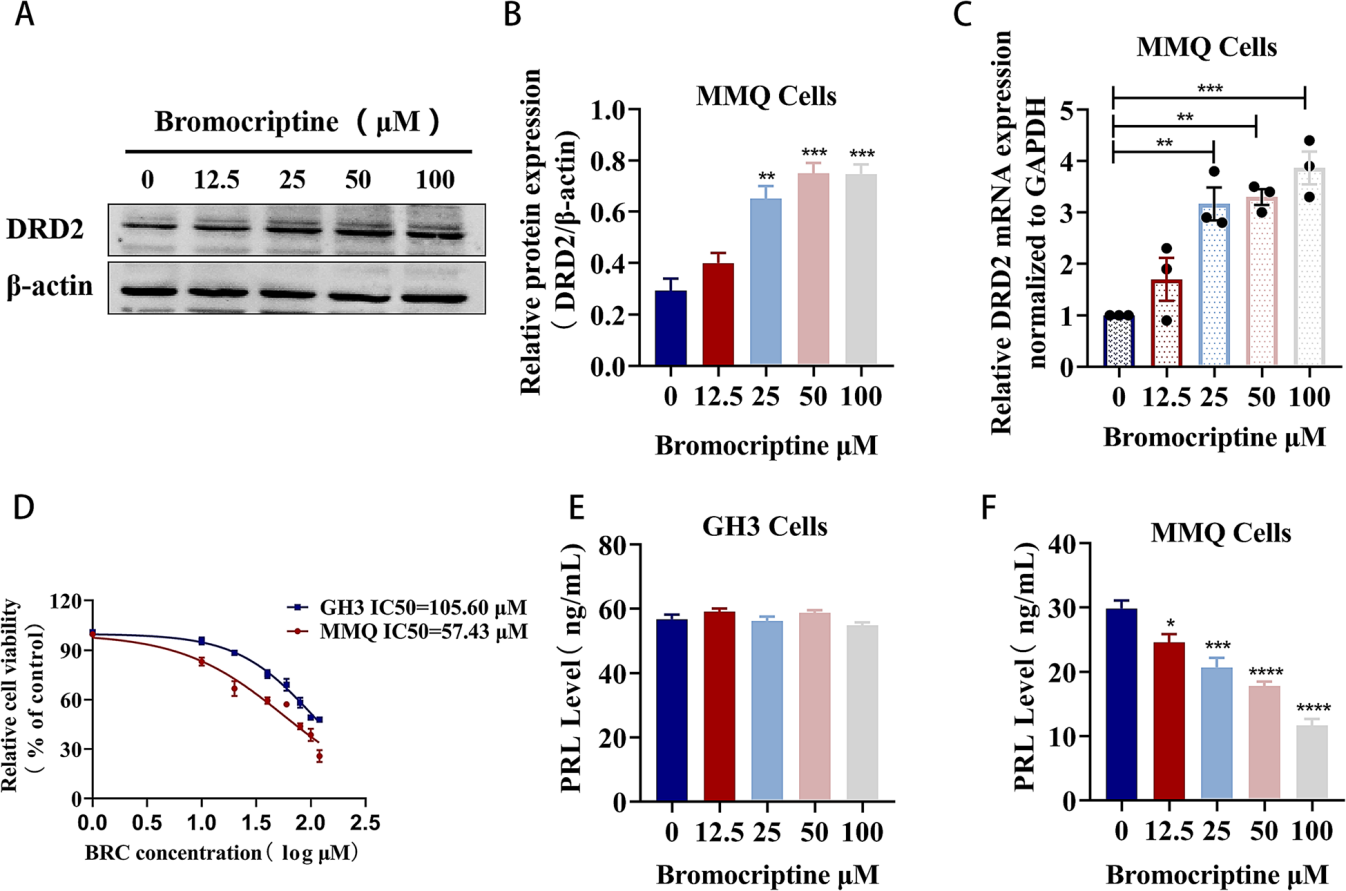
GH3 cells are resistant to bromocriptine and MMQ cells are sensitive to bromocriptine. **a**, **b** Western Blot analysis of the DRD2 protein expression level of different concentrations of bromocriptine (12.5 μM, 25 μM, 50 μM, 100 μM) treated MMQ cells for 48 h.^**^P < 0.01 vs. 0 μM,^***^P < 0.001 vs. 0 μM, (*n* = 3). **c** RT-qPCR analysis of DRD2 mRNA expression levels in MMQ cells treated with different concentrations of bromocriptine(12.5 μM, 25 μM, 50 μM, 100 μM) for 48 h.^**^P < 0.01 vs. 0 μM,^***^P < 0.001 vs. 0 μM, (*n* = 3). **d** CCK-8 kit detects the cell viability of bromocriptine on MMQ and GH3 cells. **e**, **f** The expression of PRL protein in the supernatant of GH3 and MMQ cells treated with bromocriptine for 48 h was detected by ELISA.^*^p < 0.05 vs. 0 μM,^***^p < 0.001 vs. 0 μM,^****^p < 0.0001 vs. 0 μM, (*n* = 6)

MMQ cells were treated with different concentrations of bromocriptine (12.5 μM, 25 μM, 50 μM, and 100 μM) for 48 h. Western blot results showed that bromocriptine significantly upregulated the expression of DRD2 protein (*P* < 0.01) (Fig. 2a, b) and mRNA (*P* < 0.01) (Fig. 2c) in MMQ cells. The CCK-8 kit was used to detect the viability of MMQ and GH3 cells after treatment with bromocriptine. As shown in Fig. 2d, the IC50 values for GH3 cells and MMQ cells were 105.6 μM and 57.43 μM, respectively. The IC50 value of bromocriptine in GH3 cells was much greater than that in MMQ cells, and its IC50 value was 1.8 times that of MMQ cells. After treating MMQ cells and GH3 cells with bromocriptine (12.5 μM, 25 μM, 50 μM, and 100 μM), bromocriptine significantly inhibited the PRL level in the supernatant of MMQ cells in a dose-dependent manner (*P* < 0.05) (Fig. 2f), while bromocriptine had no effect on GH3 cells (Fig. 2e). The above experimental results indicate that GH3 cells are resistant to bromocriptine and that MMQ cells are sensitive to it, which may be related to the lack of dopamine D2 receptor or low dopamine D2 receptor expression in GH3 cells and the existence of D2R in MMQ cells.

### MAPK14 and MAPK11 mediated bromocriptine resistance in prolactinomas

**Fig. 3:**
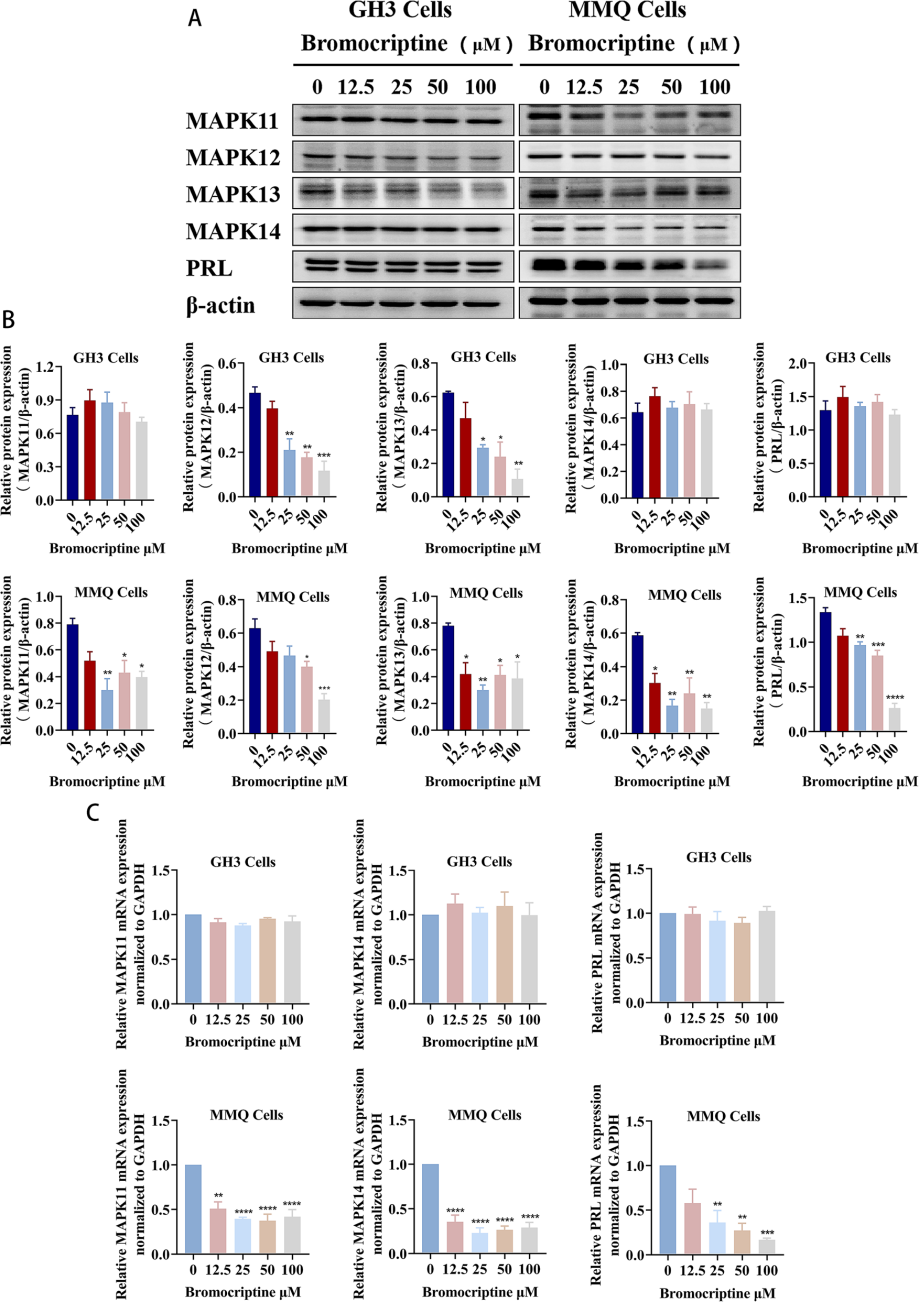
Comparison of the effects of bromocriptine on MAPK11/12/13/14 and PRL in GH3 cells and MMQ cells. **a**, **b** Western blot analysis of the protein expression of MAPK11, MAPK12, MAPK13, MAPK14 and PRL after treating GH3 cells and MMQ cells with different concentrations of bromocriptine(12.5 μM, 25 μM, 50 μM, 100 μM) for 48 h.^*^p < 0.05 vs. 0 μM,^**^p < 0.01 vs. 0 μM,^***^p < 0.001 vs. 0 μM,^****^p < 0.0001 vs. 0 μM, (*n* = 3). **c** RT-qPCR analysis of MAPK11, MAPK14 and PRL mRNA expression after bromocriptine treatment of GH3 cells and MMQ cells for 48 h.^**^p < 0.01 vs. 0 μM,^***^p < 0.001 vs. 0 μM,^****^p < 0.0001 vs. 0 μM, (*n* = 3)

After bromocriptine treatment of MMQ cells and GH3 cells for 48 h, western blotting and RT-qPCR results showed that bromocriptine significantly decreased the PRL protein level (*P* < 0.01) (Fig. 3a, b) and PRL mRNA level (*P* < 0.01) (Fig. 3c) in MMQ cells, while it had no effect on the PRL protein level (Fig. 3a, b) or PRL mRNA (Fig. 3c) level in GH3 cells. Bromocriptine significantly reduced the protein expression levels of MAPK12 (*P* < 0.05) and MAPK13 (*P* < 0.05) in GH3 and MMQ cells (Fig. 3a, b). Bromocriptine significantly reduced the protein (Fig. 3a, b) and mRNA levels (Fig. 3c) of MAPK14 and MAPK11 in MMQ cells (*P* < 0.05) but had no remarkable effect on these levels in GH3 cells. Bromocriptine had no effect on MAPK14 and MAPK11 levels in DA-resistant GH3 cells, while it significantly reduced MAPK14 and MAPK11 levels in DA-sensitive MMQ cells, indicating that MAPK11 and MAPK14 may be involved in one of the mechanisms of drug resistance in prolactinomas.

### Bromocriptine resistance in prolactinomas is related to the apoptosis signalling pathway

**Fig. 4:**
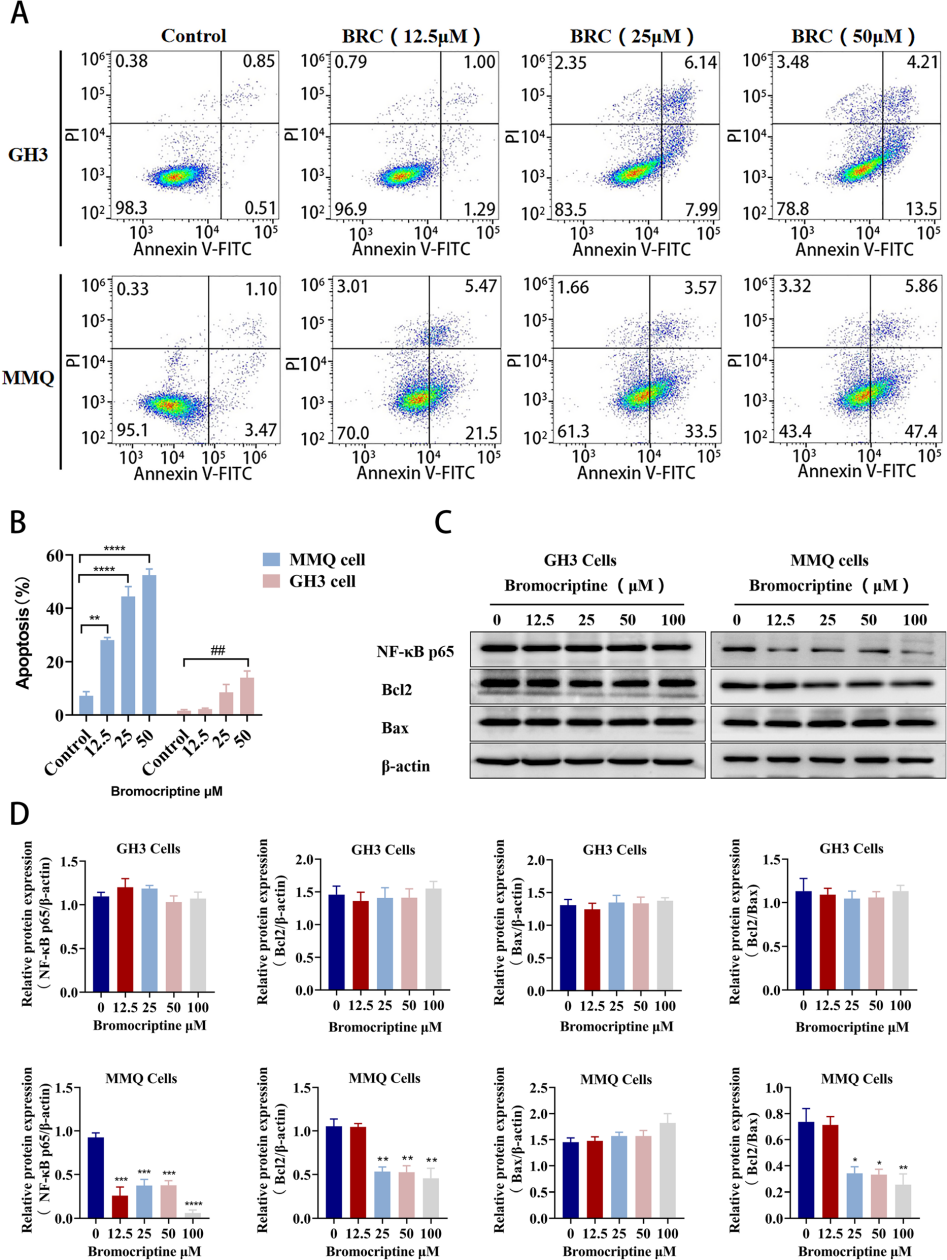
Comparison of the effects of bromocriptine on NF-κB p65, Bcl2 and Bax in GH3 cells and MMQ cells. **a**, **b** The apoptosis rate of GH3 and MMQ cells treated with bromocriptine (12.5 μM, 25 μM, 50 μM) for 48 hours was detected by Annexin V-FITC and PI double staining.^**^p < 0.01 vs. Control,^***^p < 0.001 vs. Control, ^****^p < 0.0001 vs. control, (*n* = 3). **c**, **d** Western blot analysis of NF-κB p65, Bcl2 and Bax protein expression after bromocriptine treatment of GH3 cells and MMQ cells for 48 h.^**^p < 0.01 vs. 0 μM,^***^p < 0.001 vs. 0 μM,^****^p < 0.0001 vs. 0 μM, (*n* = 3)

After MMQ and GH3 cells were treated with bromocriptine at different concentrations (12.5 μM, 25 μM, and 50 μM), cell apoptosis was detected by flow cytometry. The results showed that bromocriptine could induce apoptosis in both GH3 and MMQ cells, but the same concentration of bromocriptine had a significantly higher apoptotic rate on MMQ cells than GH3 cells, and the change trend of bromocriptine-induced MMQ cell apoptosis was more significant than that of GH3 cells (Fig. 4a, b). Furthermore, MMQ and GH3 cells were treated with different concentrations of bromocriptine (12.5 μM, 25 μM, 50 μM, and 100 μM) for 48 h. We found that bromocriptine significantly reduced the protein expression of NF-κB p65 (*P* < 0.001) and Bcl2 (*P* < 0.01) in MMQ cells but had no effect on the protein expression of NF-κB p65 and Bcl2 in GH3 cells (Fig. 4c, d). Bromocriptine had no effect on the protein expression of Bax in the two cell lines, but compared with the control group, the Bcl2/Bax protein level ratio was significantly decreased in the bromocriptine-administered MMQ cells (*P* < 0.05) (Fig. 4d). In comparison, bromocriptine significantly reduced the expression of NF-κB p65 and Bcl2 in bromocriptine-sensitive prolactinoma cells, while it had little effect on the expression of NF-κB p65 and Bcl2 in bromocriptine-resistant prolactinoma cells. Therefore, bromocriptine-resistant prolactinomas are associated with the NF-κB p65/Bcl2/Bax apoptosis signalling pathway.

### Downregulation of MAPK14 protein reverses the resistance of GH3 cells to bromocriptine

**Fig. 5:**
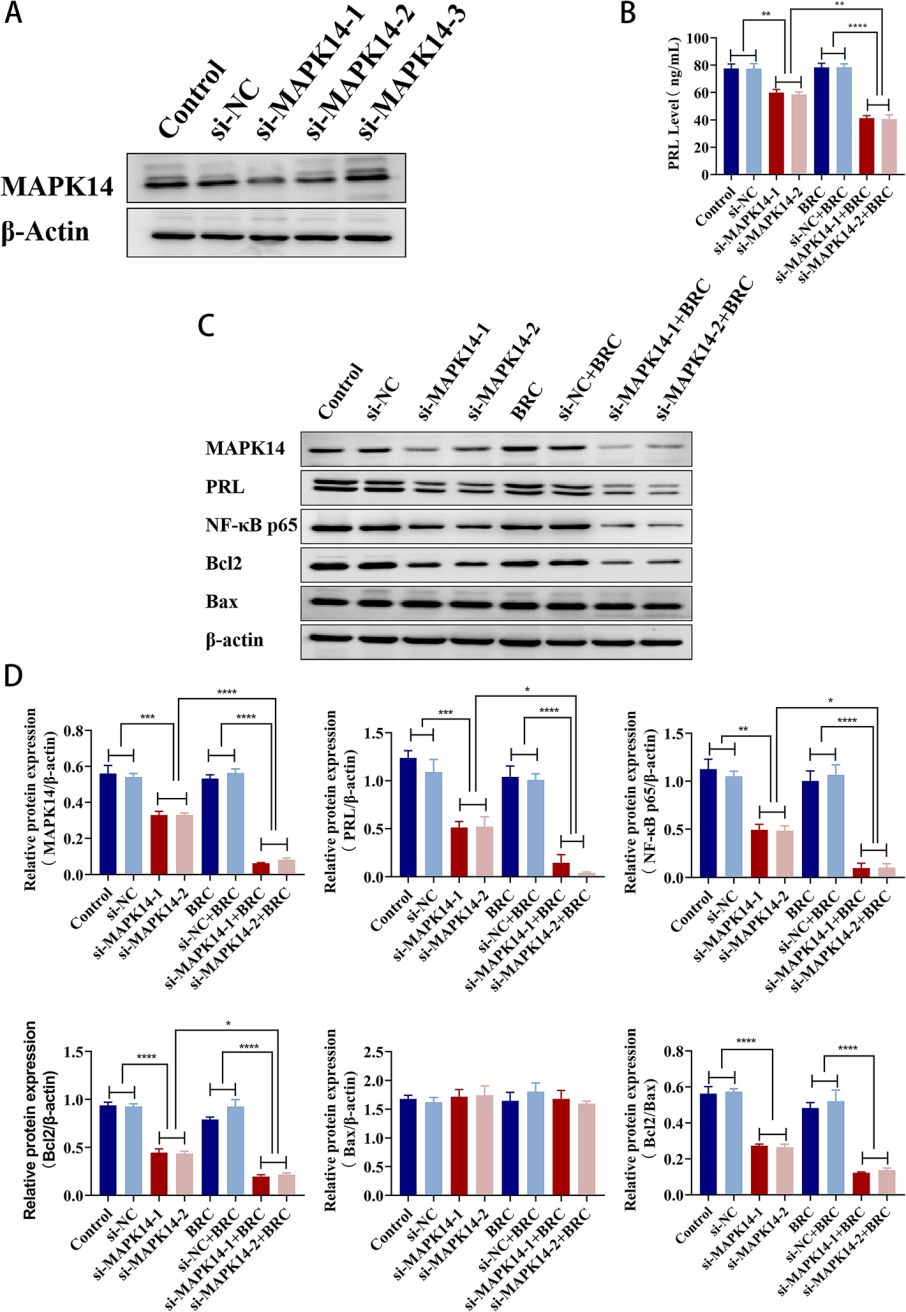
MAPK14 siRNA was transfected into GH3 cells to detect the effect of MAPK14 protein on the expression of PRL, NF-κB p65, Bcl2 and Bax in GH3 cells regulated by bromocriptine. **a** Western blotting was used to analyse the knockdown efficiency of three MAPK14 siRNA (100 nM) transfected GH3 cells for 72 h. **b** The level of PRL in the supernatant of GH3 cells was detected by ELISA.^**^p < 0.01, ^****^p < 0.0001 (*n* = 6). **c**, **d** The protein expression levels of PRL, NF-κB p65, Bcl2 and Bax in GH3 cells were analysed by western blotting.^*^p < 0.05, ^**^p < 0.01, ^***^p < 0.001,^****^p < 0.0001 (*n* = 3)

After 72 h of transfection with three different MAPK14 siRNAs (100 nM) in GH3 cells, we found that the knockdown efficiency of MAPK14 siRNA-1 and MAPK14 siRNA-2 was relatively better (Fig. 5a). GH3 cells were transfected with MAPK14 siRNA (100 nM) for 24 h and then treated with or without bromocriptine ( 50 μM) for 48 h. The level of PRL in the cell supernatant was detected by ELISA, and the protein expression was detected by western blotting. Compared with the control group and the si-NC group, after GH3 cells were transfected with siMAPK14, the expression level of PRL in the cell supernatant was significantly decreased (*P* < 0.01) (Fig. 5b), and the protein expression levels of MAPK14 (*P* < 0.001), PRL (*P* < 0.001), NF-κB p65 (*P* < 0.01), Bcl2 (*P* < 0.0001) and the ratio of Bcl2/Bax protein expression(*P* < 0.0001) were significantly decreased (Fig. 5c, d). The expression level of PRL in the cell supernatant was significantly decreased (*P* < 0.0001) (Fig. 5b), and the protein expression levels of MAPK14, NF-κB p65, Bcl2, PRL and the Bcl2/Bax protein expression level ratio were significantly decreased (*P* < 0.0001) (Fig. 5c, d) in GH3 cells transfected with siMAPK14 and treated with bromocriptine, compared with the BRC group and the si-NC + BRC group. Compared with the si-MAPK14 group, the expression level of PRL in the cell supernatant was significantly decreased (*P* < 0.01) (Fig. 5b), and the protein expression levels of MAPK14 (*P* < 0.0001), PRL (*P* < 0.05), NF-κB p65 (*P* < 0.05) and Bcl2 (*P* < 0.05) were significantly decreased (Fig. 5c, d) in GH3 cells transfected with siMAPK14 and treated with bromocriptine. In conclusion, knocking down MAPK14 in GH3 cells downregulated the protein levels of NF-κB p65, Bcl2, and PRL in GH3 cells. Bromocriptine treatment of GH3 cells transfected with MAPK14 siRNA enhanced the inhibitory effect of bromocriptine on NF-κB p65, Bcl2 and PRL. The results showed that downregulation of MAPK14 in GH3 cells can promote apoptosis and thus reverse the resistance of GH3 cells to bromocriptine.

### Downregulation of MAPK11 protein reverses bromocriptine resistance in GH3 cells

**Fig. 6:**
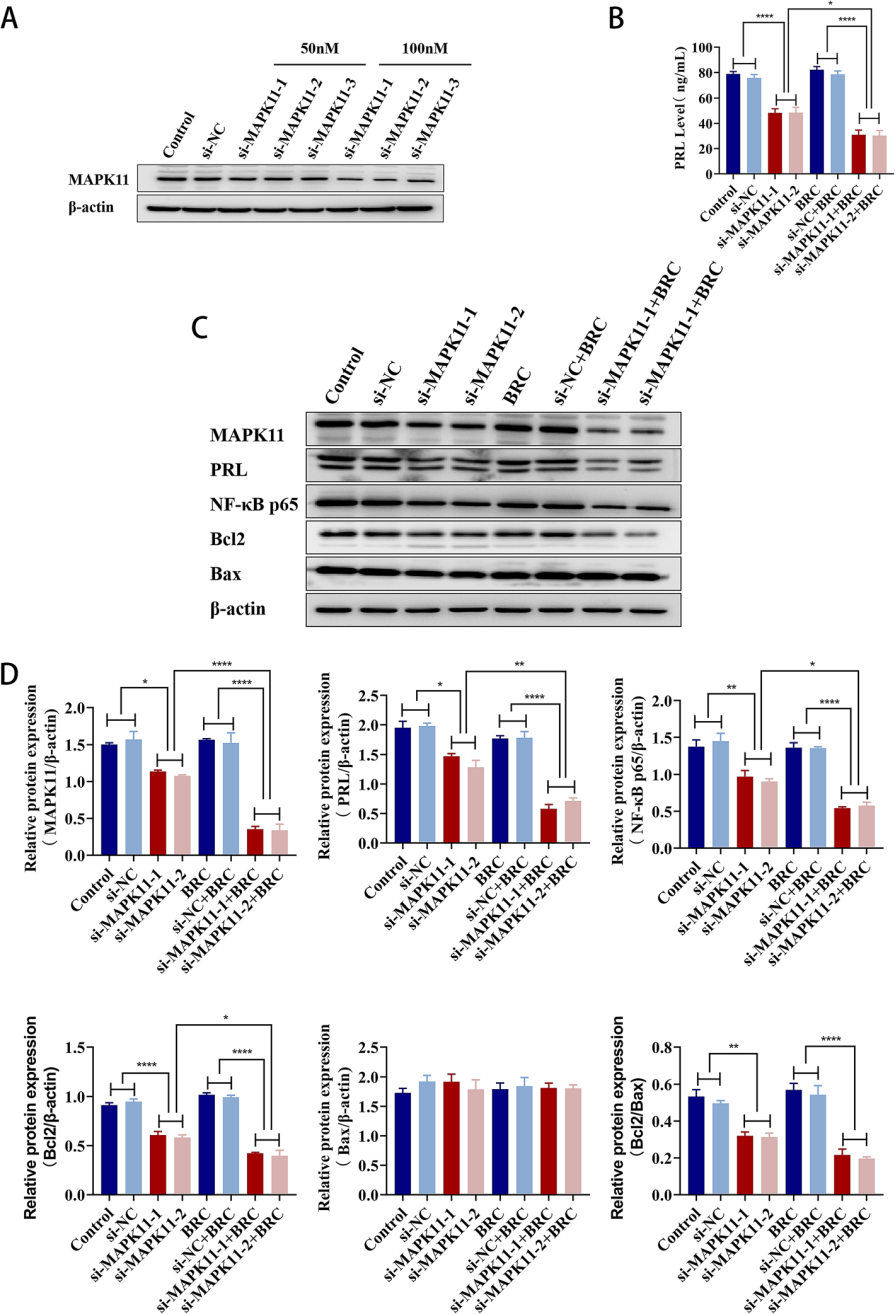
MAPK11 siRNA was transfected into GH3 cells to detect the effect of MAPK11 protein on the expression of PRL, NF-KB p65, Bcl2 and Bax in GH3 cells regulated by bromocriptine. **a** Western blotting was used to analyse the knockdown efficiency of three MAPK11 siRNA (50 nM, 100 nM) transfected into GH3 cells for 72 h. **b** The level of PRL in the supernatant of GH3 cells was detected by ELISA.^*^p < 0.05, ^****^p < 0.0001 (*n* = 6). **c**, **d** The protein expression levels of PRL, NF-κB p65, Bcl2 and Bax in GH3 cells were analysed by western blotting.^*^p < 0.05, ^**^p < 0.01, ^****^p < 0.0001 (*n* = 3)

Three MAPK11 siRNAs (50 nM, 100 nM) were transfected into GH3 cells for 72 h, and results showed that 100 nM of MAPK11 siRNA-1 and MAPK11 siRNA-2 had better knockdown efficiency (Fig. 6a). GH3 cells were transfected with MAPK11 siRNA (100 nM) for 24 h and then treated with or without BRC (50 μM) for 48 h. The PRL level of the cell supernatant was detected by ELISA, and the protein expression was detected by western blotting. Compared with the control and si-NC groups, after GH3 cells were transfected with siMAPK11, the expression level of PRL in the cell supernatant was significantly decreased (*P* < 0.0001) (Fig. 6b), and the protein expression levels of MAPK11 (*P* < 0.05), PRL (*P* < 0.05), NF-κB p65 (*P* < 0.01), and Bcl2 (*P* < 0.0001) and the protein expression ratio of Bcl2/Bax (*P* < 0.01) were significantly decreased (Fig. 6c, d). Compared with the BRC group and the si-NC + BRC group, after GH3 cells were transfected with siMAPK11 and treated with bromocriptine, the expression level of PRL in the cell supernatant was significantly decreased (*P* < 0.0001) (Fig. 6b), and the protein expression levels of MAPK11, NF-κB p65, Bcl2, and PRL and the protein expression ratio of Bcl2/Bax were significantly decreased (*P* < 0.0001) (Fig. 6c, d). Compared with the si-MAPK11 group, GH3 cells transfected with siMAPK11 were treated with bromocriptine, the expression level of PRL in the cell supernatant was significantly decreased (*P* < 0.05) (Fig. 6b), and the protein expression levels of MAPK11 (*P* < 0.0001), PRL (*P* < 0.01), NF-κB p65 (*P* < 0.05) and Bcl2 (*P* < 0.05) were significantly decreased (Fig. 6c, d). Knockdown of MAPK11 had the same effect as knockdown of MAPK14 in GH3 cells, which downregulated the protein levels of NF-κB p65, Bcl2, and PRL in GH3 cells. Bromocriptine treatment of GH3 cells transfected with MAPK11 siRNA enhanced the inhibitory effect of bromocriptine on NF-κB p65, Bcl2, and PRL. The results showed that downregulation of MAPK11 in GH3 cells can promote apoptosis and thus reverse the resistance of GH3 cells to bromocriptine.

### Bromocriptine regulates the expression of MAPK11 or MAPK14 by upregulating the expression level of DRD2 in MMQ cells, thereby reducing the expression of PRL in MMQ cells

**Fig. 7:**
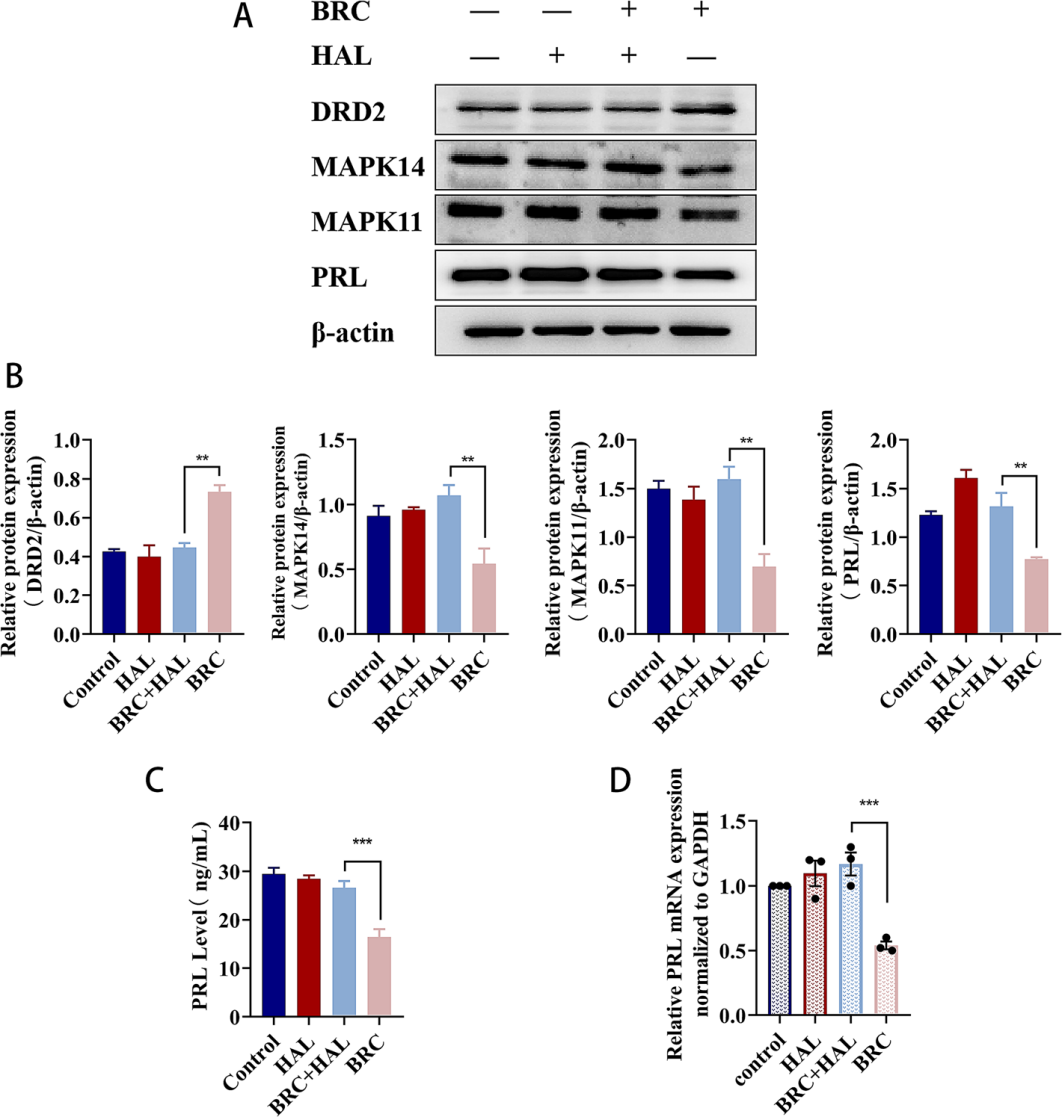
Haloperidol blocks the effect of bromocriptine on MMQ cells. **a**, **b** Western blot analysis of DRD2, MAPK14, MAPK11 and PRL protein expression in MMQ cells pretreated with haloperidol for 4 h with or without bromocriptine for 48 h.^**^p < 0.01 (*n* = 3). **c** The expression of PRL in the supernatant of MMQ cells was detected by ELISA.***p < 0.001 (*n* = 6). **d** The expression of PRL mRNA in MMQ cells was detected by RT-qPCR assays.***p < 0.001 (*n* = 3)

MMQ cells were pretreated with the dopamine antagonist haloperidol (15 μM) for 4 h and then treated with or without the dopamine agonist bromocriptine (25 μM) for 48 h [35, 36]. Western blotting results showed that compared with the combined administration group, the DRD2 protein level in the bromocriptine (BRC) administration alone group was significantly increased (*P* < 0.01), but the protein levels of MAPK14, MAPK11, and PRL in the bromocriptine (BRC) administration alone group were significantly decreased (*P* < 0.01) (Fig. 7a, b). However, there was no statistically significant difference in the expression levels of proteins in the HAL group and combined administration group compared with the control group (Fig. 7a, b). Compared with the combined administration group, the level of PRL in the cell supernatant (*P* < 0.001) (Fig. 7c) and the expression of PRL mRNA in the cells (*P* < 0.001) (Fig. 7d) of the bromocriptine (BRC) administration alone group were significantly reduced. Haloperidol blocks the agonistic effect of bromocriptine on dopamine D2 receptors and blocks the inhibitory effects of bromocriptine on MAPK11, MAPK14, and PRL. Bromocriptine regulates the expression of MAPK11 or MAPK14 by upregulating the expression level of DRD2 in MMQ cells, thereby reducing the expression of PRL in MMQ cells.

## Discussion

Pituitary adenoma, a type of benign neoplasm, is a common problem in neurosurgery and endocrinology [37–39]. Bromocriptine and cabergoline are the first choices to treat prolactinoma, as they can rapidly shrink tumour size, improve compression and reduce the PRL level [40, 41]. However, some patients are selectively resistant to DAs, and the total prevalence of bromocriptine and cabergoline resistance in prolactinoma is 20–30% and 10%, respectively [42]. Drug-resistant prolactinoma is mainly related to DA receptor regulation, but the exact molecular mechanism of bromocriptine resistance in prolactinoma is unclear. At present, there is still no effective solution for bromocriptine resistance in prolactinomas. There are several reports showing that p38 MAPK participates in multidrug resistance in breast cancer [43], ovarian cancer [44], head and neck squamous cell carcinoma [45], colon cancer [46], nasopharyngeal cancer [47], and gastric cancer [48]. Inhibition of p38 can reduce the drug resistance of the tumours mentioned above and restore drug sensitivity [49]. At present, we know little about the mechanism of the four p38 MAPK subtypes in prolactinoma and their role in DA-resistant prolactinomas.

In this study, we first reported the role of MAPK11, MAPK12, MAPK13, and MAPK14 in DA-resistant prolactinoma. Oestrogen-induced rat prolactinoma not only has high induction efficiency but also has good stability; it is an extremely ideal animal model for studying the pathogenesis and efficacy of prolactinoma [31–34]. We established a prolactinoma rat model by intraperitoneal injection of oestradiol and administered bromocriptine. The results showed that bromocriptine can treat rat prolactinomas by upregulating DRD2 and downregulating MAPK11/12/3/14, which provides a theoretical basis for the follow-up study of bromocriptine-resistant prolactinomas. GH3 cells are resistant to DAs due to the lack of dopamine D2 receptors or low dopamine D2 receptor expression, while MMQ cells are sensitive to DAs [27–30]. We used this as a theoretical basis to study the relationship between the four p38MAPK subunits and bromocriptine resistance in prolactinomas. Bromocriptine had no effect on MAPK12 and MAPK13 in either GH3 or MMQ cells, while it had a more sensitive effect on MAPK11 and MAPK14 in MMQ cells than in GH3 cells. The amino acid sequence similarity between p38α and p38β is approximately 75%, and that between p38γ and p38δ is approximately 70% [50], suggesting that MAPK11, MAPK12, MAPK13, and MAPK14 are all involved in the occurrence and development of prolactinoma. MAPK12 and MAPK13 may play a consistent role in the occurrence and development of prolactinoma. In addition, MAPK14 and MAPK11 play the same role in the occurrence and development of prolactinoma, and they synergistically participate in bromocriptine-resistant prolactinoma.

Currently, studies have shown that bromocriptine can induce apoptosis by regulating the p38 MAPK pathway and then treat pituitary tumours [51]. The transcription factor NF-κB, responsible for the regulation of cell apoptosis, can be activated by P38 MAPK and further participate in multidrug resistance in tumours [52–55]. Our previous studies also indicated that the MAPK/NF-κB signalling pathway is involved in the occurrence and development of prolactinoma [56]. We found that bromocriptine had no effect on the expression of NF-κB p65, Bcl2 and Bax in GH3 cells, while it significantly reduced the expression of NF-κB p65 and Bcl2 in MMQ cells, suggesting that NF-κB p65-mediated apoptosis is involved in bromocriptine-resistant prolactinomas and that the MAPK11, MAPK14, NF-κB p65, and Bcl2/Bax cascade pathways are associated with DA-resistant prolactinomas. To further study the interaction of MAPK11/14 with NF-κB p65 and Bcl2/Bax and its relationship with the drug resistance of prolactinoma, transfection of MAPK11 siRNA or MAPK14 siRNA into GH3 cells decreased MAPK11 or MAPK14 expression in drug-resistant GH3 cells. The reduction of MAPK11 or MAPK14 expression in GH3 cells reversed the effect of bromocriptine on NF-κB p65 and Bcl2, and the resistance of GH3 cells to bromocriptine was also reversed. These results shows that MAPK11 and MAPK14 regulate the cell apoptosis pathway and are one of the drug resistance mechanisms of prolactinomas.

Bromocriptine resistance in prolactinoma is often accompanied by downregulated D2R [57, 58]. However, there is no evidence for a direct link between bromocriptine resistance and downregulated D2R expression, and thus, the specific mechanism of bromocriptine-resistant prolactinoma due to downregulated D2R still needs to be further studied. In this study, bromocriptine showed no effect on MAPK11, MAPK14 and PRL in GH3 cells lacking DRD2, but it significantly reduced these proteins in MMQ cells with DRD2. Studies have shown that DA inhibits the MAPK signalling pathway, thereby inhibiting prolactin secretion and the proliferation of prolactin cells [59, 60]. DA-mediated apoptosis shows apparent obligate involvement of p38 MAPK and/or ERK in cells that express either endogenous or transfected D2R receptors. At the same time, some studies have shown that dopamine does not significantly activate p38 MAPK or JNK in GH3 cells and that DA-mediated apoptosis is mediated through the DA transporter (DAT). Other studies have shown that dopamine does not activate MAPKs but induces apoptosis. GH3 cells do not express a functional D2 receptor and are resistant to DA-mediated apoptosis, but their sensitivity is restored in cells transfected with a functional D2R [61], which is consistent with our findings. Bromocriptine induced apoptosis in GH3 cells, with concomitant activation of p38 MAPK [62]. D2R overexpression could affect the MAPK pathway, prolactin cell proliferation and PRL secretion to varying degrees [7]. There were different results on the specific mechanism by which D2R regulates p38MAPK. Is the drug resistance of prolactinoma regulated by MAPK14/11 also associated with DRD2? Our results show that antagonizing DRD2 in MMQ cells can block the inhibitory effects of bromocriptine on MAPK11, MAPK14, and PRL in MMQ cells, suggesting that DRD2-mediated MAPK11 and MAPK14 may participate in DA resistance in prolactinoma. It is suggested that DRD2 participates in DA resistance in prolactinoma by mediating MAPK11 and MAPK14. Our research group is continuing to study the specific regulatory relationship between DRD2 and MAPK11/14.

## Conclusion

**Fig. 8:**
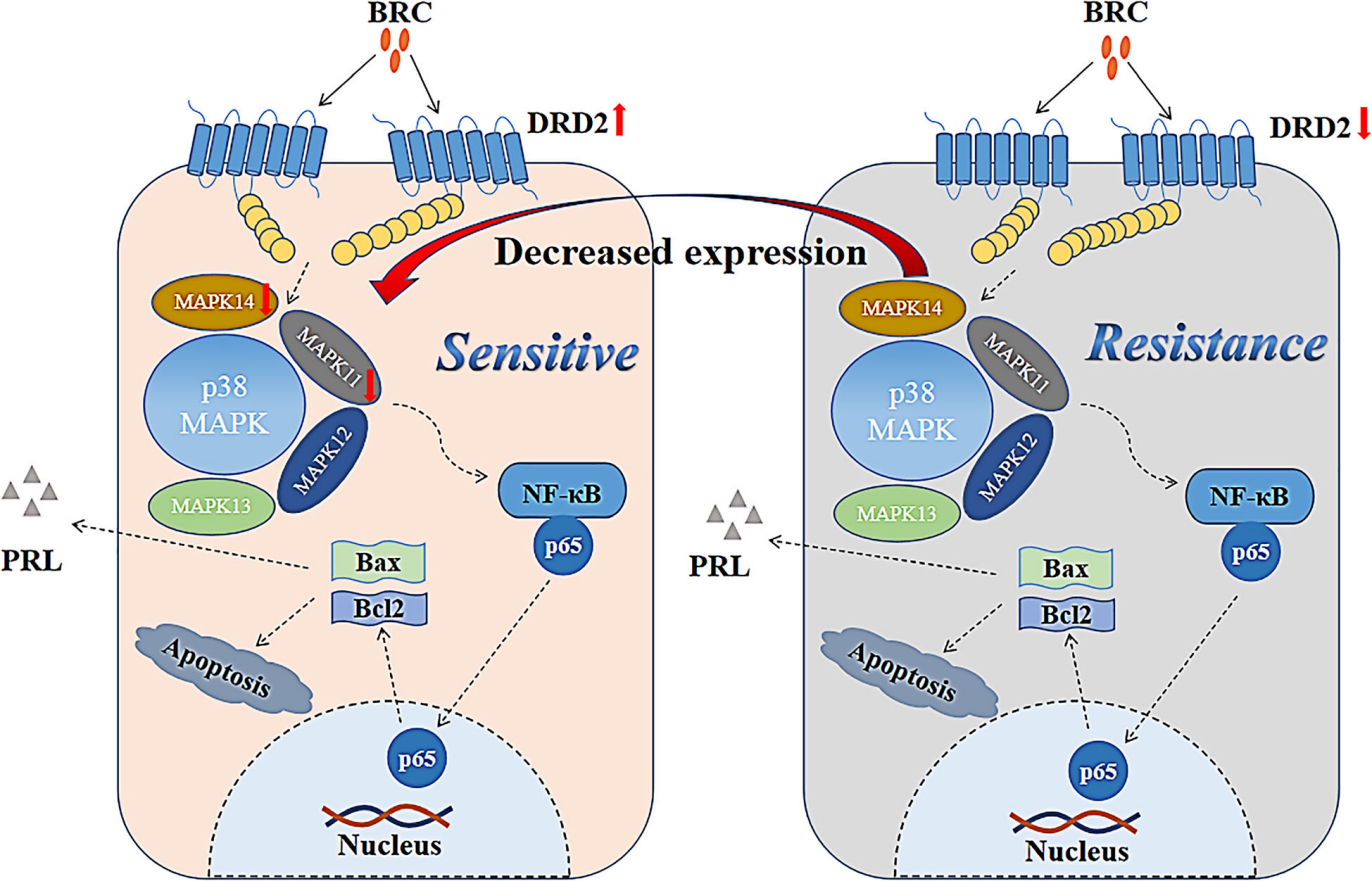
Molecular mechanism of bromocriptine resistance in prolactinomas. Bromocriptine binds to DRD2 on the surface of prolactin cells, upregulates the expression of DRD2, downregulates the expression of MAPK11 and MAPK14, promotes cell apoptosis, and inhibits PRL secretion, thereby reversing the bromocriptine resistance of prolactinomas and restoring the sensitivity of prolactinoma to bromocriptine. The lack or low expression of DRD2, bromocriptine had no significant effect on MAPK11 and MAPK14 proteins, leading to bromocriptine resistance in prolactinomas

In summary, this is the first study to confirm that bromocriptine can treat prolactinoma by reducing MAPK11, MAPK12, MAPK13, and MAPK14. The molecular mechanism of bromocriptine-resistant prolactinomas is shown in Fig. 8. MAPK11 and MAPK14 mediate bromocriptine resistance in prolactinomas by regulating cell apoptosis. Reduction of MAPK11 and MAPK14 expression can reverse bromocriptine resistance in prolactinomas. Therefore, MAPK11 and MAPK14 may be novel targets for the treatment of DA-resistant prolactinoma.

## Data Availability

All data generated or analyzed during this study and supporting our findings are included and can be found in the manuscript. The raw data can be provided by corresponding author on reasonable request.

## Abbreviations

BRC: Bromocriptine
HAL: Haloperidol
PRL: Prolactin
MAPK: Mitogen-activated protein kinase
DAs: Dopamine agonist
DRD2: Dopamine D2 receptor
siRNA: Small interfering RNA
ELISA: Enzyme-Linked Immunosorbent Assay
RT-qPCR: Real-time fluorescence quantitative PCR
